# Feasibility of spondyloplasty with SpineJack^®^ implants in patients with thin vertebral pedicles

**DOI:** 10.1007/s00330-026-12719-w

**Published:** 2026-07-09

**Authors:** Marion Bouchet, Jean-Baptiste Pialat, Bastien Chalamet, William Boulade, Charles Mayard, Adrian Kastler, Sylvain Grange, Nicolas Stacoffe

**Affiliations:** 1 Department of Diagnostic and Interventional Radiology, Hopital Lyon Sud, Hospices Civils de Lyon, 69310 Oullins-Pierre-Bénite, France; 2 Department of Diagnostic and Interventional Radiology, Centre Hospitalier Edouard Herriot, Hospices Civils de Lyon, 69003 Lyon, France; 3 Diagnostic and Interventional Neuroradiology Unit, Centre Hospitalier Universitaire de Grenoble, 38700 La Tronche, France; 4 Department of Radiology, Hôpital Nord, Centre Hospitalier Universitaire de Saint-Etienne, 42270 Saint-Priest en Jarez, France

**Keywords:** Vertebral augmentation, Spondyloplasty, Interventional radiology, SpineJack®

## Abstract

**Objective:**

To evaluate the feasibility of spondyloplasty with SpineJack^®^ implants in patients with thin vertebral pedicles, outside the manufacturer’s recommendation.

**Materials and methods:**

In this retrospective bicentric study, all patients treated with spondyloplasty using SpineJack^®^ implants for traumatic, porotic or malignant vertebral compression fractures were enrolled. Pedicle diameter was assessed to classify patients into the “Thin Pedicle” (TP) group, outside the manufacturer’s recommendation (< 5 mm and/or safety margin < 0.8 mm between implant and pedicle), or into the “Advised Pedicle Size“ (APS) group. The primary outcome was correct implant positioning on postoperative imaging. Secondary outcomes included restoration of vertebral height, procedure duration, radiation dose, hospital stay, and complications.

**Results:**

A total of 148 patients (mean age 49 ± 37 years ; 55% of male) underwent 151 spondyloplasties: 51 in the TP group and 100 in the APS group. Correct implant positioning was achieved in all procedures in both groups (100%). There was no significant difference in vertebral height restoration (81% vs. 71%; *p* = 0.058). Radiation exposure was significantly lower in the TP group (*p* = 0.02), while procedure duration and hospital stay were comparable, with 55% of procedures performed on an outpatient basis. Complication rates did not differ significantly, consisting mostly of cement leakages (20% vs. 19%; *p* = 0.93).

**Conclusion:**

Spondyloplasty with SpineJack^®^ implants under radiological guidance appears feasible and safe in patients with thin pedicles, even when the pedicle size is below the manufacturer’s suggested threshold.

**Key Points:**

***Question*** *Does pedicle size below recommended thresholds affect procedural feasibility, safety, and clinical outcomes when performing SpineJack® implantation under radiological guidance?*

***Findings**** SpineJack® implantation achieved 100% correct positioning in thin pedicles, with complication rates, procedure duration, and hospital stay comparable to standard pedicle sizes*.

***Clinical relevance**** These findings support expanding SpineJack® indications to patients with narrow pedicles, increasing access to safe and efficient minimally invasive vertebral fracture treatment for a broader patient population*.

**Graphical Abstract:**

**Figure:**
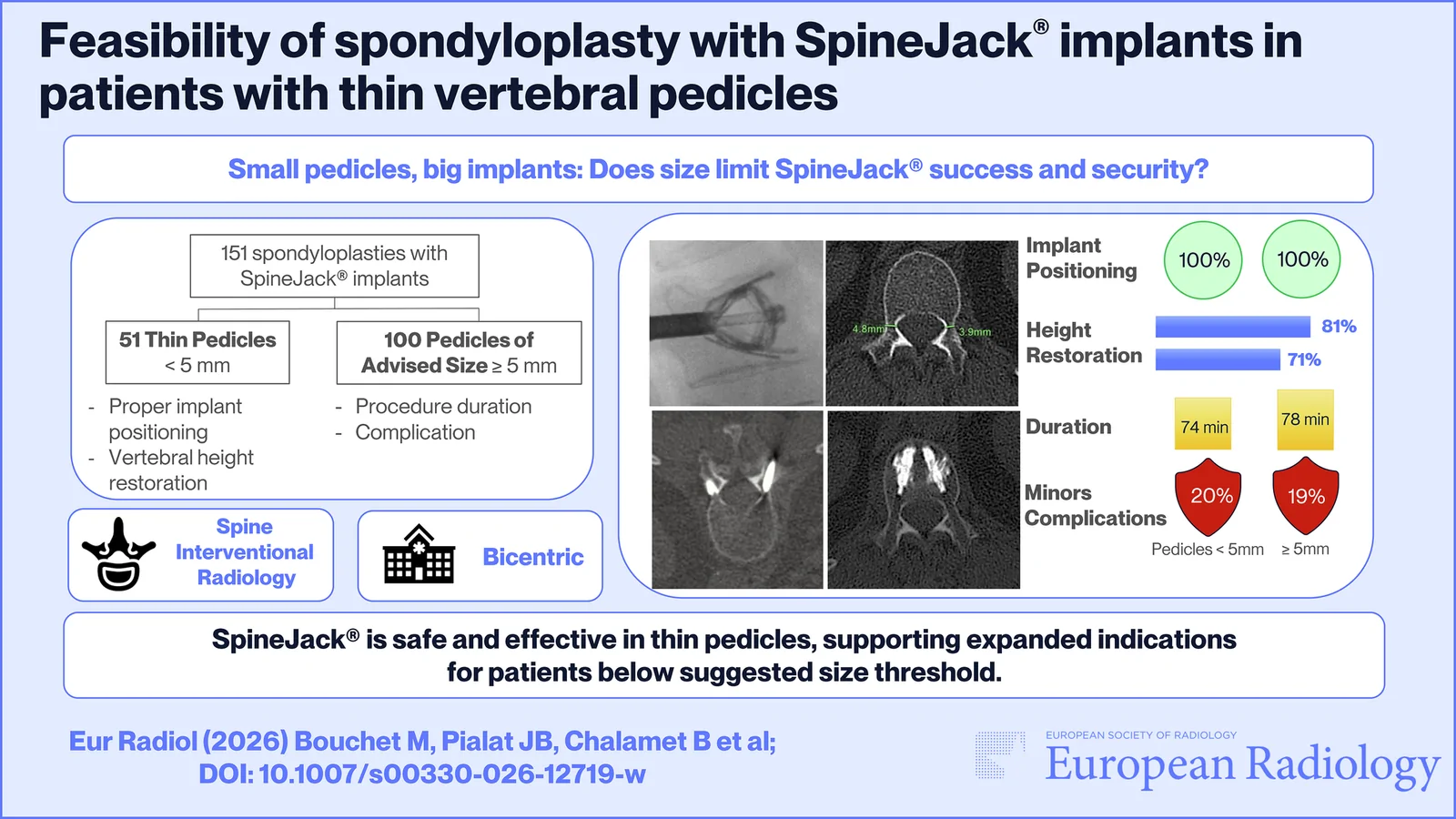

## Introduction

Vertebral compression fracture is a common condition, which typically occurs through two main mechanisms in distinct populations: traumatic fractures in younger patients, and osteoporotic fractures in the elderly. Among the available therapeutic options, percutaneous vertebroplasty has been used for decades as a minimally invasive technique that allows rapid and effective pain relief through the injection of polymethylmethacrylate cement (PMMA) [1–3]. While effective in stabilizing the fractured vertebra, vertebroplasty has no effect on the deformity induced by the collapse of the vertebral body. This can contribute to persistent postural imbalance, progression of local kyphosis and adjacent-level fractures [4–7].

To overcome these limitations, vertebral body expansion techniques have been developed. Among them, implant-based systems like SpineJack^®^ (Stryker) have demonstrated superior outcomes compared to balloon kyphoplasty, particularly in vertebral height restoration and kyphosis correction [8, 9]. This titanium alloy device is inserted percutaneously and expanded to elevate the endplates and restore vertebral anatomy. This technique improves the biomechanical outcome of vertebroplasty, while remaining a minimally invasive procedure [10–13].

The manufacturer advises a minimum pedicle diameter of 5 mm to ensure safe implant insertion, maintaining a safety margin of 0.8 mm between the pedicle and the implant diameter. This threshold of 5 mm is commonly reported in the literature [10, 12–14]. However, in our practice, we have performed this procedure in patients with narrower pedicles, adapting the approach and assessing the feasibility and safety of this extended strategy. We present the results of a 3-year retrospective feasibility study.

## Materials and methods

This was a bicentric retrospective study approved by the institutional ethics committee (identifier 25-5255), where all participants provided informed consent.

### Study population

All patients who underwent percutaneous spondyloplasty with the SpineJack^®^ implant at the two interventional radiology centers of our institution between November 2021 and March 2025 were included in the study.

Patients were excluded if no pre- or post-operative imaging was available to obtain vertebral dimensions or if they refused the use of their data.

Data were retrospectively collected from medical records, including age, sex, body mass index, procedural and hospitalization details (procedure duration, radiation dose, anesthesia type, imaging guidance modality, and length of hospital stay). Vertebral and fracture characteristics (dimensions, height loss, etiology, AO Spine classification) were independently collected and assessed from pre-operative CT scans by a junior radiologist and a senior radiologist specialized in interventional musculoskeletal imaging. Vertebral height was measured at the anterior, middle, and posterior portions, and the mean value of these three measurements was used for analysis. Measurements were performed on both pre-operative CT scans and immediate post-procedural imaging to assess vertebral height restoration [15].

Pedicle measurements were performed on pre-operative scans to identify patients with pedicle diameters outside the manufacturer’s recommendation (< 5 mm and/or a margin between the pedicle and implant diameters < 0.8 mm). Patients with narrower pedicles were classified into the “Thin Pedicle group” (TP group), while those with pedicles of suggested size formed the “Advised Pedicle Size group” (APS group).

### Implant selection

The SpineJack^®^ implant is available in three sizes, defined by their unexpanded diameters: 4.2, 5.0, and 5.8 mm. The manufacturer suggests choosing the implant based on vertebral anatomical characteristics, while maintaining a safety margin of 0.8 mm between the pedicle and the implant diameter.

The maximum cranio-caudal expansion varies from 12.5 mm for the smallest model to 20.0 mm for the largest model, with lift forces ranging from 500 to 1000 N during expansion.

The implant size was selected before the spondyloplasty procedure, based on pre-operative CT scans, taking into account pedicle diameter, vertebral body length and height loss.

### Spondyloplasty procedure

The procedure was performed under general anesthesia with the patient in a prone position. Prophylactic antibiotic treatment with 2 g of intravenous cefazolin was administered to reduce the risk of infection [16].

Procedures were performed either in a CT-scanner suite equipped with a mobile fluoroscopic C-arm or in an interventional radiology suite with cone-beam CT (CBCT) navigation (Azurion Flex, Philips) [17, 18]. The choice of guidance modality depended on equipment availability and hospital protocols.

**Fig. 1:**
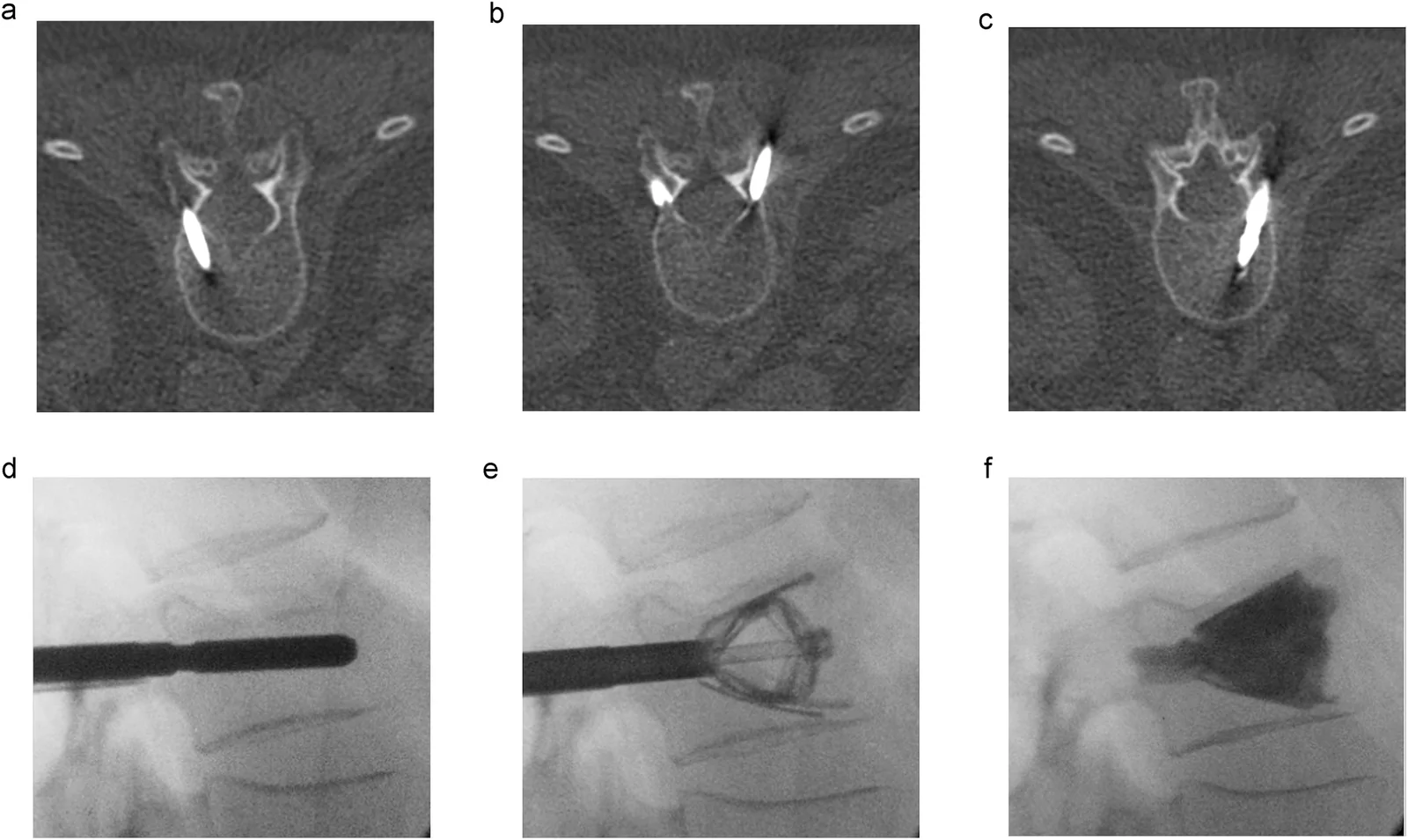
SpineJack^®^ implant procedure under scanner and fluoroscopic guidance. **a**, **b** Insertion of the cannula via a bilateral transpedicular approach. **c** Advancement of the reamer to the anterior third of the vertebral body. **d** Insertion of the template to verify implant length. **e** Implant expansion. **f** Final control after cement injection

The standardized procedure consisted of a bilateral pedicular approach to the fractured vertebra, using an access cannula to insert the guidewire and advance the reamer up to the anterior third of the vertebral body. A template was used to prepare the implant path and verify sufficient length for implant placement. The unexpanded implant was then inserted and progressively deployed under fluoroscopic guidance to restore vertebral height. Finally, cement (PMMA, Vertaplex® HV) was injected under real-time fluoroscopy to fill the vertebral body and secure the implant (Fig. 1).

### Radiological assessment

At least three CT or CBCT acquisitions were systematically performed during the procedure: an initial scan to plan the implant trajectory, a second scan to verify correct template alignment, and a final scan to confirm proper implant positioning and detect potential complications.

### Primary and secondary outcomes

#### Primary outcome

The primary outcome was proper positioning of the implant within the vertebral body, assessed on post-procedural CT or CBCT imaging. Implant placement was considered correct in the absence of anterior, posterior, pedicular, or discal protrusion, according to the manufacturer’s recommendations.

#### Secondary outcomes

Secondary outcomes included:

- Vertebral height restoration is estimated by comparing pre- and post-procedural vertebral body heights. Theoretical vertebral height was defined as the average height of the adjacent superior and inferior vertebrae. When post-procedural vertebral height exceeded the theoretical height, the percentage of height restoration was greater than 100%.
- Procedure duration (in minutes) and radiation dose (in mGy·cm²), as reported in the operative record.
- Length of hospital stay (in days), from admission to discharge.

Peri-procedural complications were evaluated on final imaging, with a focus on cement leakages, categorized as intravenous, intradiscal, epidural, or paravertebral [19].

### Statistical analysis

Analyses were performed using R version 4.4.1. Quantitative variables were expressed as mean ± standard deviation and compared between groups using Student’s *t*-test (Welch’s correction applied by default in case of unequal variances). Qualitative variables were reported as counts and percentages and compared using Pearson’s *χ*² test or Fisher’s exact test when expected frequencies were < 5. Statistical significance was defined as a *p*-value < 0.05. A multivariate analysis was performed to identify independent factors associated with procedure duration, radiation dose, and vertebral height restoration.

## Results

### Patient characteristics

A total of 149 patients were initially retrieved; one patient was excluded due to a missing preoperative CT scan. Thus, 148 patients were included in the analysis, corresponding to 151 spondyloplasties. Among them, 51 vertebrae had pedicles considered too thin according to the manufacturer’s recommendation (thin pedicle, “TP” group), while 100 had pedicles within the advised size range (advised pedicle size, “APS” group). The procedures were performed by one of 12 radiologists specialized in musculoskeletal interventional imaging.

The overall sex ratio was 1.22, with male patients representing 55% of the cohort. A significantly higher proportion of female patients was observed in the TP group (61% vs. 39%, *p* = 0.01).

**Fig. 2:**
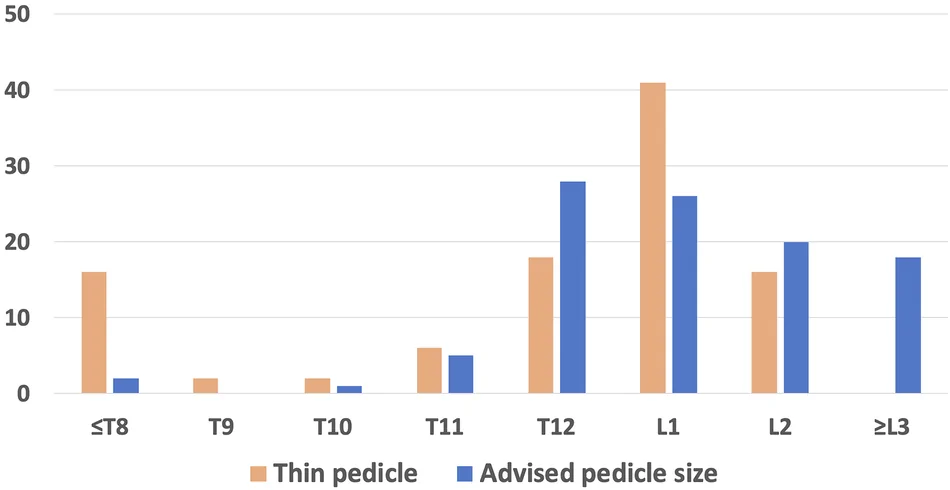
Distribution of fractured vertebral level

Fractures were most frequently located at the thoracolumbar junction, a region subject to high mechanical stress [20]. Patients in the TP group were significantly more likely to present upper thoracic fractures (above T10; *p* < 0.001; Fig. 2). The majority of fractures in both groups were classified as AO Spine type A1, with 47 patients presenting A2, A3, or A4 fractures, all without neurological impairment or indications for neurosurgical intervention [14, 21, 22].

The interval between fracture and procedure was shorter in the TP group (9 days vs. 12 days; *p* = 0.03). Patients in the TP group also had a significantly lower BMI than those in the APS group (23.8 ± 3.7 vs. 25.4 ± 4.8 kg/m², *p* = 0.02). No significant differences were observed between groups regarding other variables, including fracture etiology (traumatic, osteoporotic or malignant; *p* = 0.99), vertebral height loss (28% vs. 30%; *p* = 0.37) or polytrauma (defined as the presence of at least one additional severe injury such as multiple fractures, pelvic fractures, flail chest, or traumatic brain injury; *p* = 0.53).

All demographic and baseline characteristics are summarized in Table 1.

**Table 1:** Patient and fracture characteristics

	Total	Thin pedicle	Advised pedicle size	*p*-value for the difference between groups
	*n* = 151	*n* = 51	*n* = 100	
Patient characteristics				
Age	49 ± 19	47 ± 19	51 ± 18	0.22^a^
Sex				0.01^b^
Female	68 (45%)	31 (61%)	37 (37%)	
Male	83 (55%)	20 (39%)	63 (63%)	
BMI (kg/m^2^)	24.7 ± 4.5	23.8 ± 3.7	25.4 ± 4.8	0.02^a^
Fracture characteristics				
Pedicle diameter (mm)				----^c^
Left pedicle	6.6 ± 1.8	4.6 ± 0.8	7.6 ± 1.4	
Right pedicle	6.6 ± 1.9	4.8 ± 0.9	7.5 ± 1.5	
Fracture-to-procedure interval (days)	11 ± 10	9 ± 4	12 ± 12	0.03^a^
Fractured vertebrae				< 0.001^b^
≤ T10	13 (9%)	10 (20%)	3 (3%)	
T11	8 (5%)	3 (6%)	5 (5%)	
T12	37 (24%)	9 (18%)	28 (28%)	
L1	47 (31%)	21 (40%)	26 (26%)	
L2	28 (19%)	8 (16%)	20 (20%)	
≥ L3	18 (12%)	0	18 (18%)	
AO Spine classification				0.50^b^
A1	104 (69%)	34 (67%)	70 (70%)	
A2	11 (7%)	2 (4%)	9 (9%)	
A3	32 (21%)	13 (25%)	19 (19%)	
A4	4 (3%)	2 (4%)	2 (2%)	
Height loss (%)	29 ± 13	28 ± 12	30 ± 13	0.37^a^
Fracture etiology				0.99^b^
Traumatic	128 (85%)	43 (84%)	85 (85%)	
Porotic	20 (13%)	7 (14%)	13 (13%)	
Malignant	3 (2%)	1 (2%)	2 (2%)	
Polytrauma	54 (36%)	16 (31%)	38 (38%)	0.53^b^

Values are presented as the number of patients (%) or mean ± SD

^a^ Welch’s *t*-test

^b^ Chi-square test

^c^ Pedicle diameter was the group-defining variable and was therefore not tested

### Primary outcome

**Fig. 3:**
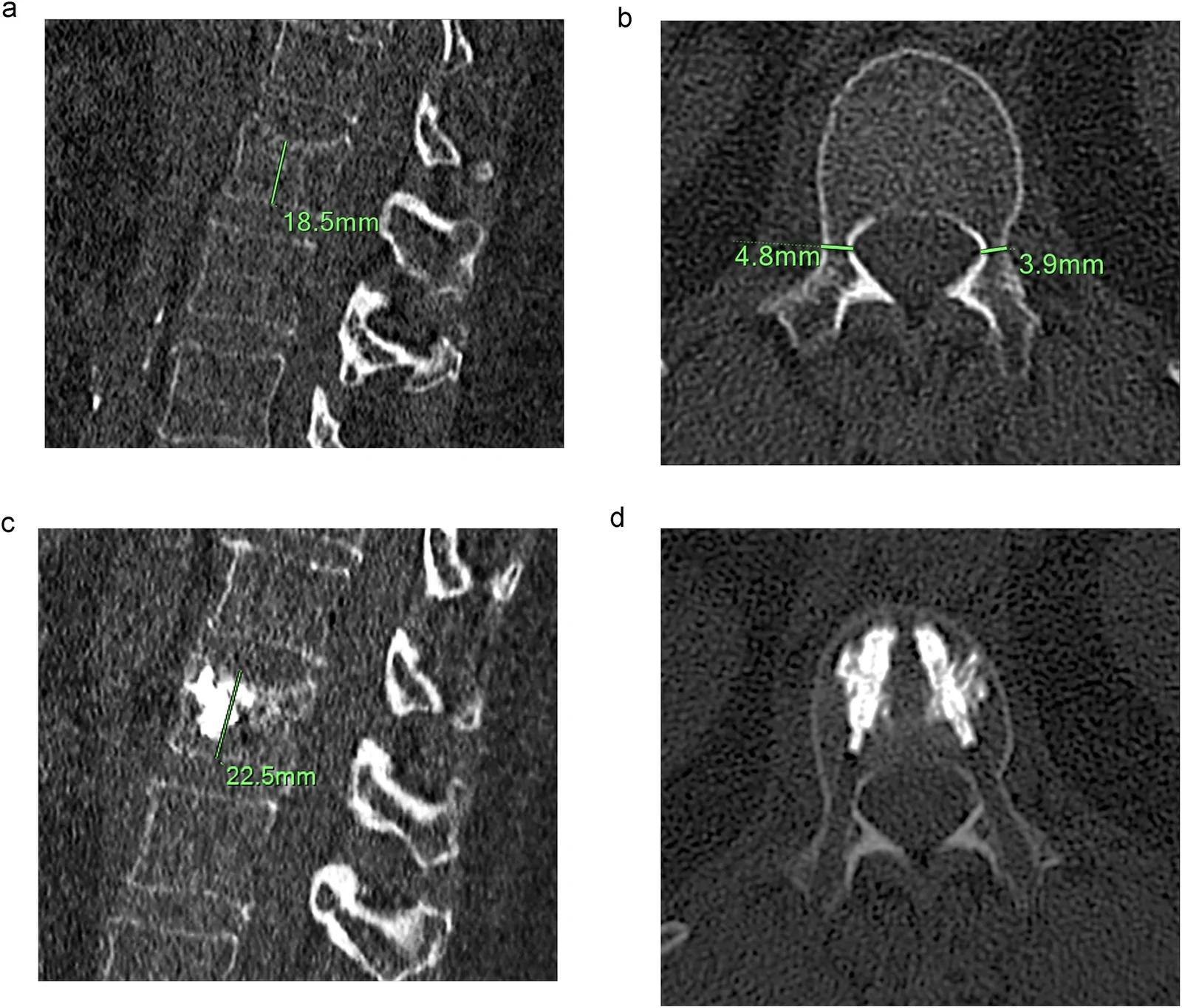
52-year-old female patient with a traumatic AO-Spine-A1 L1 fracture, without neurological symptoms. Preoperative CT-scan (**a**, **b**) showed thin pedicles (4.8 and 3.9 mm). Spondyloplasty with SpineJack^®^ was performed under CT guidance. A 4.2 mm implant was chosen. The height restoration was 75%, without complication (**c**, **d**)

Proper positioning of the SpineJack^®^ implant was achieved in 100% of cases, including in the 51 patients of the TP group (Fig. 3).

The 5.0-mm implant was the most frequently used in both groups (73% vs. 71%). As expected, the smaller 4.2-mm implant was more often selected in the TP group (22% vs. 4%), whereas the larger 5.8-mm implant was predominantly used in the APS group (25% vs. 6%; *p* < 0.001).

### Secondary outcomes

The median vertebral height restoration was 74% ± 31, with no significant difference between the groups (81% vs. 71%; *p* = 0.06).

Radiation exposure, however, was significantly lower in the TP group (22,208 vs. 31,101 mGy.cm^2^ ; *p* = 0.02). Procedure duration (74 vs. 78 min) was comparable between groups (*p* = 0.37). The mean length of hospital stays (3 days; range: 0–55) was also similar (*p* = 0.27), with 55% of patients treated on an outpatient basis.

All the results are summarized in Table 2.

**Table 2:** Overview of the results

	Total	Thin pedicle	Advised pedicle size	*p*-value
Results	*n* = 151	*n* = 51	*n* = 100	
SpineJack^®^ diameter				< 0.001^a^
0.4.2	15 (10%)	11 (22%)	4 (4%)	
0.5.0	108 (72%)	37 (72%)	71 (71%)	
0.5.8	28 (18%)	3 (6%)	25 (25%)	
Proper positioning	151 (100%)	51 (100%)	100 (100%)	
Vertebral height restoration (%)	74 ± 31	81 ± 31	71 ± 30	0.06^b^
Procedure duration (min)	77 ± 26	74 ± 26	78 ± 26	0.37^b^
Radiation dose (mGy.cm^2^)	31101 ± 35979	22208 ± 27068	35787 ± 39209	0.02^b^
Length of hospital stay (days)	3 ± 6	2 ± 4	3 ± 7	0.27^b^
Complications	29 (20%)	10 (20%)	19 (19%)	0.93^a^
Cement leakage	28 (19%)	9 (18%)	19 (19%)	
Intravenous	11 (7%)	5 (10%)	6 (6%)	
Intradiscal	4 (3%)	0	4 (4%)	
Epidural	3 (2%)	0	3 (3%)	
Paravertebral	10 (7%)	4 (8%)	6 (6%)	
Other complications	1 (1%)	1 (2%)	0	
Guidance				0.42^a^
CBCT	91 (60%)	33 (65%)	58 (58%)	
CT-scan	60 (40%)	18 (35%)	42 (42%)	
Combined procedures	39 (26%)	11 (22%)	28 (28%)	0.42^a^

Values are presented as the number of patients (%) or mean ± SD

^a^ Chi-squared test

^b^ Welch’s *t*-test

Subgroup analyses were performed to identify independent factors associated with procedure duration, radiation dose, and vertebral height restoration. A significant interaction was observed between height restoration and the fracture-to-procedure interval: an interval < 7 days was associated with greater height recovery (+ 2%; *p* < 0.001). Logically, combined procedures (defined as the addition of vertebroplasty or pelvic osteosynthesis following spondyloplasty) were associated with longer operative times (+ 33 min; *p* < 0.001) and higher radiation doses (+ 463.6 mGy·cm²; *p* < 0.01). No significant associations were found for age or fracture etiology.

### Complications

Complications were rare. Cement leakage was observed in 19% of patients (*n* = 28), with no significant difference between groups (18% vs. 19%; *p* = 0.93). The majority of leaks were minor venous or paravertebral. These events were classified as grade 1 according to the Clavien-Dindo classification of surgical complications [23].

Only one complication unrelated to cement leakage was reported: a per-procedural pneumothorax in a patient in the TP group, caused by pleural recess puncture during the initial vertebral approach, requiring drainage (grade 3b of the Clavien-Dindo classification). The patient was discharged the day after the procedure.

No pedicle fracture was observed in either group.

## Discussion

Thin pedicles are often considered a limiting factor for the use of SpineJack^®^ implants, with a pedicle diameter threshold of 5 mm. This bicentric retrospective study aimed to assess whether the procedure remains feasible and safe in this population.

In our cohort of 151 percutaneous spondyloplasties, 34% of cases involved a pedicle diameter of less than 5 mm and/or a margin of less than 0.8 mm between the pedicle and implant diameters. Nevertheless, the implant was correctly positioned in 100% of procedures. These findings suggest that the technical challenges associated with thin pedicles can be effectively managed in routine clinical practice through careful preoperative planning and precise imaging guidance.

 Implant placement in patients with thin pedicles was not associated with a higher complication rate, cement leakage, or other complications (*p *= 0.93). Thus, a reduced pedicle diameter does not appear to compromise procedural safety.

The overall cement leakage rate was 19% across the cohort, with most cases involving minor paravertebral or venous leaks. This is consistent with previously published data on the subject [13, 24, 25].

Regarding efficacy, vertebral height restoration was higher in the TP group (81% vs. 71%; *p* = 0.06), although the difference did not reach statistical significance. The fracture-to-procedure interval was shorter in this group (9 vs. 12 days, *p* = 0.03), suggesting that less consolidated fractures may have a greater potential for height recovery. Subgroup analysis confirmed this trend, showing that earlier treatment was significantly associated with improved vertebral height restoration (*p* < 0.001).

Procedure times were comparable between groups, with no significant difference observed, in agreement with a previous study [17]. Radiation exposure was lower in the TP group (*p* = 0.02), which may be partly explained by lower BMI and the use of optimized imaging protocols in younger patients, in whom minimizing irradiation is prioritized. The proportion of combined procedures was similar across groups and therefore did not appear to influence overall procedure duration or radiation exposure.

There was a significantly higher proportion of upper thoracic vertebrae (above T10) in the TP group (30% vs. 3%, *p* < 0.001). This is consistent with vertebral anatomy, as pedicles are narrower in the upper thoracic spine than at lower thoracic and lumbar levels [26, 27]. Additionally, the higher proportion of female patients in the TP group (61% vs. 37%; *p* = 0.01) may also be relevant, since females generally exhibit smaller pedicles than males [28].

A posterior pedicular approach was preferred over parapedicular routes to reduce the risk of bleeding or cement leakage [29]. Additionally, it ensures that the posterior portion of the implant remains within the bone rather than in the paravertebral soft tissues.

Depending on the fracture pattern and the initial loss of height, it may be necessary to choose an implant one size larger than recommended to achieve sufficient vertical deployment and an adequate restoration of vertebral height. Indeed, in 17 procedures, we opted for a larger implant for this purpose, with a mean height gain of 89% in these patients, versus 74% across the cohort.

This study has several limitations. Its retrospective design did not allow for standardized clinical follow-up; the potential correlation between improved vertebral height restoration and clinical outcomes could not be assessed. However, the primary objective was to evaluate the technical feasibility and safety of the procedure in cases with thin pedicles, rather than to demonstrate clinical efficacy. Implant positioning was not evaluated with objective quantitative criteria, but procedures were performed in accordance with the manufacturer’s recommendations.

Taken together, these results suggest that the indication for SpineJack^®^ implantation should not be limited by a strict pedicle-size cutoff, provided that safety principles are observed and the technique is adapted to individual anatomy (appropriate implant size and rigorous imaging control).

## Conclusion

This study demonstrates the feasibility of performing spondyloplasties with SpineJack^®^ implants via a pedicular approach under radiological guidance, using CT or CBCT, even when the pedicle size is below the suggested threshold.

It shows that patients with vertebrae considered outside the usual criteria can still be safely treated. These findings could help expand the current indications, enabling treatment of a broader patient population, including those with upper thoracic vertebral fractures or younger individuals.

## Abbreviations

APS: Advised pedicle size group
BMI: Body mass index
CBCT: Cone-beam CT
CT: CT-scanner
PMMA: Polymethylmethacrylate cement
TP: Thin pedicle group
